# Prevalence of Liver Fluke (*Fasciola hepatica*) in Wild Red Deer (*Cervus elaphus*): Coproantigen ELISA Is a Practicable Alternative to Faecal Egg Counting for Surveillance in Remote Populations

**DOI:** 10.1371/journal.pone.0162420

**Published:** 2016-09-06

**Authors:** Andrew S. French, Ruth N. Zadoks, Philip J. Skuce, Gillian Mitchell, Danielle K. Gordon-Gibbs, Alexandra Craine, David Shaw, Stuart W. Gibb, Mark A. Taggart

**Affiliations:** 1 Environmental Research Institute, North Highland College, University of the Highlands and Islands, Castle Street, Thurso, KW14 7JD, United Kingdom; 2 Moredun Research Institute, Pentlands Science Park, Bush Loan, Penicuik, EH26 0PZ, United Kingdom; 3 Institute of Biodiversity, Animal Health and Comparative Medicine, College of Medical, Veterinary and Life Sciences, University of Glasgow, Glasgow G61 1QH, United Kingdom; 4 UHI Rural Studies Centre, North Highland College, University of the Highlands and Islands, Dale Farm, Halkirk, KW12 6UW, United Kingdom; US Geological Survey, UNITED STATES

## Abstract

Red deer (*Cervus elaphus*) are hosts of liver fluke (*Fasciola hepatica*); yet, prevalence is rarely quantified in wild populations. Testing fresh samples from remote regions by faecal examination (FE) can be logistically challenging; hence, we appraise frozen storage and the use of a coproantigen ELISA (cELISA) for *F*. *hepatica* surveillance. We also present cELISA surveillance data for red deer from the Highlands of Scotland. Diagnoses in faecal samples (207 frozen, 146 fresh) were compared using a cELISA and by FE. For each storage method (frozen or fresh), agreement between the two diagnostics was estimated at individual and population levels, where population prevalence was stratified into cohorts (e.g., by sampling location). To approximate sensitivity and specificity, 65 post-slaughter whole liver examinations were used as a reference. At the individual level, FE and cELISA diagnoses agreed moderately (κ_frozen_ = 0.46; κ_fresh_ = 0.51), a likely reflection of their underlying principles. At the population level, FE and cELISA cohort prevalence correlated strongly (Pearson’s R = 0.89, p < 0.0001), reflecting good agreement on relative differences between cohort prevalence. In frozen samples, prevalence by cELISA exceeded FE overall (42.8% vs. 25.8%) and in 9/12 cohorts, alluding to differences in sensitivity; though, in fresh samples, no significant difference was found. In 959 deer tested by cELISA across the Scottish Highlands, infection prevalence ranged from 9.6% to 53% by sampling location. We highlight two key advantages of cELISA over FE: i) the ability to store samples long term (frozen) without apparent loss in diagnostic power; and ii) reduced labour and the ability to process large batches. Further evaluation of cELISA sensitivity in red deer, where a range of fluke burdens can be obtained, is desirable. In the interim, the cELISA is a practicable diagnostic for *F*. *hepatica* surveillance in red deer, and its application here has revealed considerable geographic, temporal, sex and age related differences in *F*. *hepatica* prevalence in wild Scottish Highland red deer.

## Introduction

Red deer (*Cervus elaphus*) are a recognised host of liver fluke (*Fasciola hepatica*) throughout Europe [1–5]. In contrast to domestic ruminants, in which disease monitoring is routine, apart from a handful of species, (namely, coypu (*Myocastor coypus*) [6,7], European hare (*Lepus europaeus*) [8,9], European rabbit (*Oryctolagus cuniculus*) [8], wild boar (*Sus scrofa*) [10] and roe deer (*Capreolus capreolus*) [11]), data concerning *F*. *hepatica* in wildlife is limited to incidental observations, which is the case for red deer in the Scottish Highlands [3]. In the UK as a whole, annual diagnostic rates of fasciolosis, the parasitic disease caused by *Fasciola* spp., have increased significantly in domestic sheep and cattle since the late 1990s [12] and are predicted to continue rising [13]; hence, surveillance of wild red deer in the Scottish Highlands at this moment, is desirable.

The role of wildlife as *F*. *hepatica* reservoirs (a population that harbours macro- or microparasites that can result in infection of other species or populations) is still the subject of some discussion. Typically, species are implicated following discovery of at least two of the following: i) high *F*. *hepatica* prevalence, ii) confirmation of viable eggs in host excreta, iii) close genetic relatedness between flukes collected from the suspected reservoir and another population; and, consideration of the maintenance of the *F*. *hepatica* life cycle. For example, in the northern Netherlands, *F*. *hepatica* is present in up to 41% of hares (*L*. *europaeus*); from whom, recovered fluke specimens belong to the same genetic clades as fluke recovered from cattle in the same region [9]. Similarly, *F*. *hepatica* prevalence of 11% and 40–90% egg viability, implicate wild boar (*S*. *scrofa*) as a likely reservoir to cattle in NW Spain [10].

The prevalence of *F*. *hepatica* in Scottish Highland red deer has not been objectively quantified. The most recent parasitological survey of Scottish deer (formalin archived tissue samples collected during 1991–1997) identified “non-specific parasite related changes” in the liver tissue of 10% of the population [14], perhaps an indication of *F*. *hepatica* prevalence; and, pertinent to the present study, significantly more animals with “other non-specific” signs of liver disease (though potentially also fluke related) were found in the Highlands than in any other region [14]. Moreover, within the Highland region, environmental variation is marked in terms of topography, geochemistry, climate and land use; all of which are associated with *F*. *hepatica* infection risk [15,16]. Therefore, owing to the lack of contemporary data for deer in the region and their unknown epidemiological role (potentially associated with hill sheep), one aim of this study was to quantify *F*. *hepatica* prevalence in wild red deer within the Highlands at the sub-regional hunting estate scale.

The presence of *F*. *hepatica*, established by faecal examination (FE) (often extended to faecal egg counts; FEC), requires samples to be processed fresh (i.e., prior to freezing or desiccation which may damage/deform eggs), sedimentation time, sample staining and slide preparation, and is therefore largely impractical to apply to wild deer in remote regions such as the Scottish Highlands. Alternatively, the *F*. *hepatica* coproantigen enzyme-linked immunosorbent assay (cELISA;[17]) may be more feasible, as it is based on detection of fluke excretory-secretory antigens, which remain stable when frozen [18,19]. The cELISA is also advantageous because it requires less processing time and facilitates batch testing. To date, the performance of the cELISA has shown considerable potential for sheep and cattle diagnostics [18,20–23], and in wild boar (*S*. *scrofa*) [10]; whereas in horses, the cELISA has been attributed only 9% sensitivity and has therefore been deemed unsuitable [22]. Here, the performance of the *F*. *hepatica* cELISA is compared with FE and whole liver examination (for fluke presence) in Scottish Highland red deer, considering both epidemiological and practical characteristics of the tests. In addition, we provide new data on the prevalence and distribution of liver fluke in red deer (n = 959) from Scottish Highland hunting estates.

## Materials and Methods

Sampling took place between August 2012 and February 2014. All samples were collected by deer stalkers/gamekeepers on privately owned Scottish hunting estates (Aline, Alladale, Altnaharra, Applecross Trust, Ardnamurchan, Badanloch, Ben Loyal, Conaglen, North Harris Trust and Strathconon). The owners of these estates legally delegate their right to kill deer on their land to their employee deer stalkers/gamekeepers under the Deer Act 1991 and the Deer (Scotland) Act 1996; hence, permission to sample and indeed instruction to kill wild deer during this study was given by the land owner(s) to the head deer stalker on each estate—as such, no individual deer was killed specifically for the purposes of this research. The culling of deer is carried out during routine deer management; hence, samples constituted a by-product of this activity so did not require a licence under the Animals (Scientific Procedures) Act 1986. No animals were specifically killed for this study—as such, University ethics approval was also not required. Each deer was killed by shooting in accordance with the Deer (Firearms etc.)(Scotland) Order1985 and current “Best Practice Guidance” developed within Scotland’s deer management sector.

Faecal samples from red deer were collected into 50ml centrifuge tubes (Fisher Scientific, UK). Samples were collected directly from the rectum of 959 wild red deer that had been culled by deer stalkers working on nine estates within the Scottish Highlands; and, owing to constraints on sample volumes, 353 of these samples were used for the diagnostic method evaluation aspect of this study. Samples were taken during the annual red deer cull, which runs from 1^st^ July to 20^th^ October for males, and 21^st^ November to 15^th^ February for females. A subset of these 353 samples (n = 146) were immediately refrigerated following collection (not frozen) and analysed by FEC (within seven days)—the data from which were later converted to binary FE positives and negatives. During FEC, supernatants were also collected and frozen for subsequent cELISA analysis. The remaining samples (n = 207) were frozen at -20°C on the day of collection, and then defrosted prior to FEC analysis and supernatant preparation at a later date. In addition to faecal samples, whole livers were collected from 65 of the culled individuals on two estates (49 females, 17 from Badanloch, 32 from Altnaharra; and 16 males, all from Badanloch) and stored frozen; faecal samples from these 65 individuals form part of the aforementioned subset that were stored fresh. All faecal samples were accompanied by a datasheet containing information regarding sex, age category and spatial and temporal data. However, on 32 sampling occasions, datasheets were either not provided or did not include a date of cull; these represent 19 females (9 positive diagnoses by cELISA; 6 by FE; 11 positives in total) and 13 males (7 positive by cELISA; 4 by FE; 7 positives in total). Where temporal data were available, samples were categorised by month, year, sex and sampling estate.

### Coprological methods

Coproantigen ELISA kits (specific to *F*. *hepatica*) were used to analyse faecal samples in accordance with the manufacturer’s guidelines for sheep faeces (BioX Diagnostics, Belgium). For supernatant preparation, faecal samples were homogenised with a stainless steel spatula and 0.50 ± 0.03g sub-samples were weighed into 12ml centrifuge tubes (round-bottomed, Greiner bio-one CELLSTAR, UK) to which 2ml of the kit’s dilution buffer was added. Each tube was then vortex mixed for 3s prior to centrifuging for 10 minutes at 1000*g*. Approximately 1ml of supernatant was extracted by pipette from each centrifuge tube. This was stored at -20°C in 2ml microfuge tubes (Eppendorf, Germany) until a cELISA kit plate could be filled. Eggs of *F*. *hepatica* were counted in faeces using a sedimentation technique [20].

### Whole liver examination

Whole livers were defrosted for 24 hours prior to full visual examination. All livers were sliced into 1-2cm parallel strips with a scalpel [2]. Sliced liver was then gently squeezed to encourage fluke to slide out of bile ducts and parenchyma. In each burdened animal, there was a tendency for fluke to reside in groups within pockets of pale-coloured scarred tissue, rather than in the bile ducts. These pockets were found to contain between 1 and 13 fluke and a grey-coloured viscous fluid. Fluke from each liver were removed, rinsed with MilliQ^®^ water and frozen at -20°C or preserved in 70% ethanol for future reference. Numbers of fluke heads (noted by the presence of a ventral sucker; S1 Fig) counted in each liver were recorded and animals were considered infected if at least one fluke was found.

### Sensitivity and specificity

Whole liver examination was used as a concurrent reference standard against which to estimate sensitivity and specificity of the cELISA and FE methods. However, it is acknowledged that liver examination may be imperfect (i.e., not 100% sensitive) owing to potential losses of fluke specimens at the deer larder during carcass evisceration, or simply because fluke are missed during visual inspection. As such, our estimates of specificity are inherently negatively biased owing to apparent false (FE or cELISA) positives.

Nevertheless, liver examination is 100% specific; hence our estimates of cELISA and FE sensitivity are not biased—though we recognise that FE sensitivity estimates are related to patent infection only. Confidence intervals for sensitivity and specificity (i.e., proportions) (95% CI) were calculated using Eq 1.

$${\text{CI}}_{\text{upper},\text{ lower}}=\text{ p }\pm \;1.96\;\times \sqrt{\frac{\text{p(1-p)}}{\text{n}}}$$ (1)

For completeness, we estimate the sensitivity of the cELISA to patent infection (from FE positives) in frozen and fresh (where livers were not always available) samples. We do not use the FE negative animals for calculation of specificity, because the cELISA is designed to detect pre-patent as well as patent infection (even if debatable under field conditions [20]).

### Statistical analyses

All statistical analyses were carried out using R [24] and RStudio [25]. At the population level, the correlation between estimated cohort (e.g., sex and location specific) prevalence of infection by the cELISA and the FE methods was examined using Pearson’s R coefficient using the R{cor.test} function [24]. Owing to repeated measures/correlated binary outcomes (i.e., a single subject is diagnosed by two or more of the diagnostics), a one-sided McNemar’s **χ**^**2**^ test for paired proportions (using the R {mcnemar.test} function) was used to identify whether differences in diagnostic outcomes of the FE and cELISA (as collated in 2 × 2 contingency tables) were significant.

The agreement between the three diagnostic tests was analysed separately for fresh and frozen samples at the individual and population levels. At the individual level, Cohen’s kappa (κ) was chosen to quantify the agreement between tests [26] (see S1 Text for further detail). In addition to provision of the kappa statistic, a bias effect (i.e., the difference between methods in terms of percentage of positive/negative diagnoses; apparent relative sensitivities) and a prevalence effect (i.e., the percentage of the sampled population diagnosed as infected) were quantified [27,28] (see S1 Text for further detail). For completeness, we also quote the maximum attainable kappa, proportion of observed agreement, p_o_, and the proportion of observed positive p_pos_, and negative p_neg_, agreement. Also at the individual level, Spearman’s ρ coefficient was used to quantify the association between FEC eggs per gram (epg) and cELISA titres (ELISA units (EU) equivalent to % of positive reference standard titre).

Chi-square tests of independence were used to examine the associations between prevalence (estimated by cELISA alone) of *F*. *hepatica* infection and sampling season, sex and age class; whereby the {chisq.test} function in R [24] was used. Furthermore, significant differences at the 5% level in prevalence of *F*. *hepatica* infection between months, estates and age groups were identified using post hoc Tukey contrasts using the {glht} function in the {multcomp} package [29] applied to binomial (logit link) generalised linear models {glm} in R.

## Results

### Diagnostic agreement at the population and individual level

**Fig 1:**
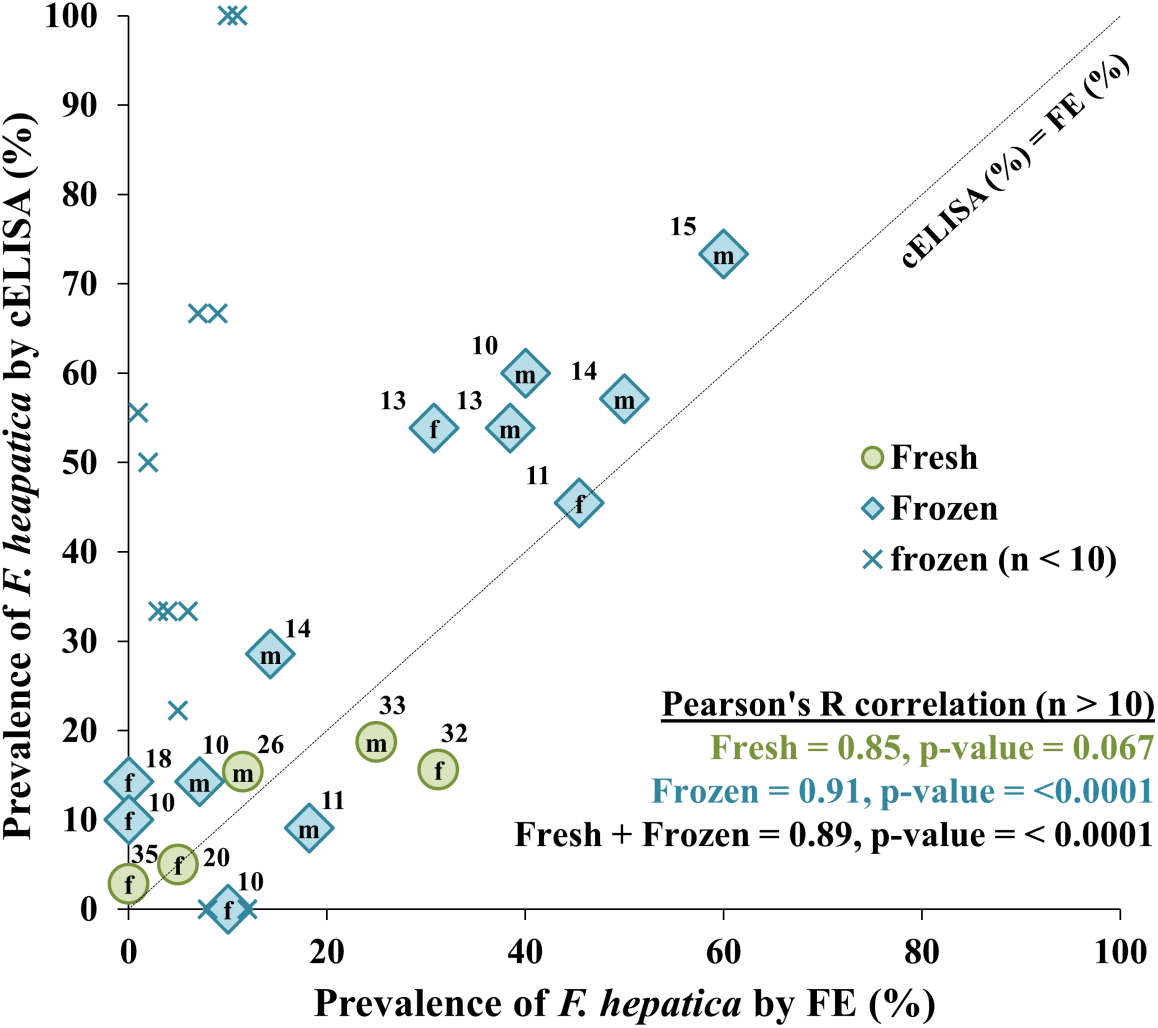
FE and cELISA estimated prevalence of *F*. *hepatica* infection (percentage of specific cohorts infected by sex (male, m; female, f) and sample storage method). Cohorts were from nine wild red deer populations during two stalking seasons (2012–13 and 2013–14); cohort sample sizes are shown next to each point. See S2 Table for details of sampling sites and seasons to which these data relate.

In 146 fresh faecal samples collected during 2013–14, prevalence of *F*. *hepatica* estimated by FE (12.3%) and cELISA (9.6%) did not differ significantly (McNemar’s χ^2^ = 0.64, df = 1, p-value = 0.42) (S1 Table); whereas in 159 frozen samples collected during 2012–13, prevalence estimated by cELISA (42.8%) was significantly greater than FE (25.8%) (McNemar’s χ^2^ = 16.5, df = 1, p-value <0.001) (S1 Table).When stratified by year, sex and month (S1 Table; where n > 10 per cohort), prevalence estimated by cELISA in frozen samples exceeded FE in 4/6 cohorts; whereas in fresh samples, prevalence estimated by cELISA only exceeded FE in 1/7 cohorts. When stratified by year, sex and estate (S2 Table; where n > 10 per cohort), prevalence in frozen samples estimated by cELISA exceeded FE in 9/12 cohorts, and estimates of prevalence by cELISA and FE were strongly correlated (Pearson’s R = 0.89, p < 0.0001) (Fig 1); whereas in fresh samples, prevalence estimated by cELISA (all 2013–14) only exceeded FE in 2/5 and the correlation between the cELISA and FE was not significant (Pearson’s R = 0.85, p < 0.067).

**Fig 2:**
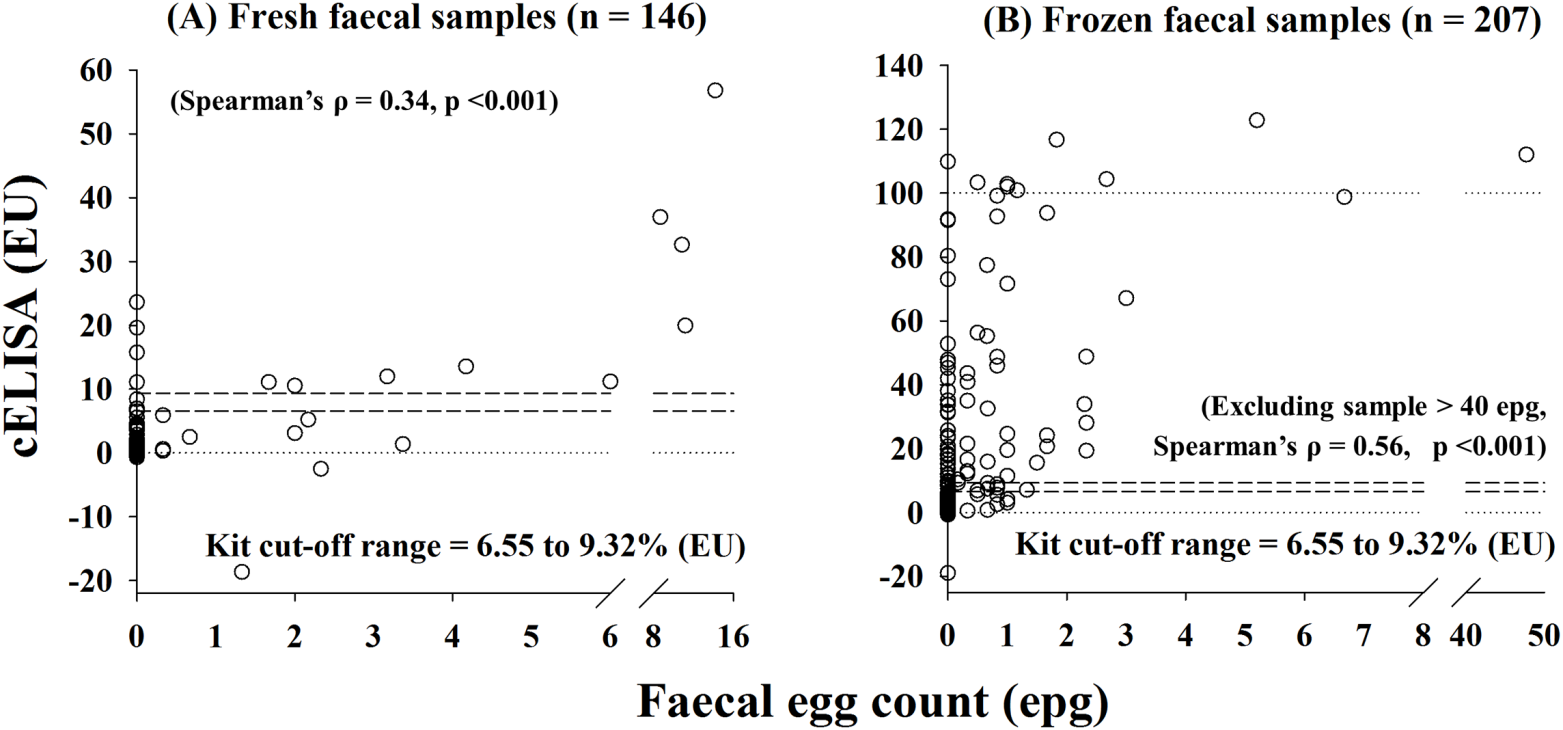
Scatter plots comparing results of FEC and a commercial cELISA (Bio X, Belgium) for *F*. *hepatica* infection in faecal samples that were collected from wild Scottish red deer between 2012 and 2014 (n = 353). Assays were carried out on samples that had been stored in two ways: (A) fresh (unfrozen) from the time of collection until time of testing (n = 146), and (B) frozen immediately after being collected from culled deer (n = 207). For the FEC test, results are recorded in eggs per gram of faeces (epg). For the cELISA, results are expressed in ELISA units (EU). Positive diagnosis by the cELISA was recorded for samples where results fell above a cut off derived using a positive reference standard.

Agreement between the cELISA and the FE at the individual level was moderate (κ_frozen_ = 0.46, κ_fresh_ = 0.51; Table 1) [30]). Of the 146 fresh faecal samples analysed, 14 were positive by the cELISA and 18 were positive by the FE method (23 positives in total)—in nine of these cases the techniques were in agreement (Table 1). Of the 207 frozen faecal samples analysed, 80 were positive by the cELISA and 52 were positive by the FE method (91 positives in total)—in 41 samples the techniques were in agreement (Table 1). In both fresh and frozen samples, there were significant correlations between FEC (epg) and cELISA (EU) (Spearman’s ρ = 0.34, p < 0.0001 for fresh; Spearman’s ρ = 0.56, p < 0.0001 for frozen) (Fig 2).

**Table 1:** Cross-tabulated comparison between the diagnostic outcomes of a cELISA (BioX, Belgium), FE and post mortem whole liver examination for *F*. *hepatica* infection using faecal samples collected from wild Scottish red deer (n = 353), stored fresh and frozen.

	**Fresh faeces (n = 146)**				**Frozen faeces (n = 207)**		
		**FE**			**FE**		
		**Positive**	**Negative**	**Total**	**Positive**	**Negative**	**Total**
**cELISA**	**Positive**	9 (*14*)	5 (*0*)	14	41 (*52*)	39 (*28*)	80
	**Negative**	9 (*4*)	123 (*128*)	132	11 (*0*)	116 (*127*)	127
	**Total**	18	128	146	52	155	207
	Agreement	9	123	132	41	116	157
	Exp. by chance (nearest whole number)	2	116	118	20	95	115
	Kappa (max attainable)	**0.51**	**(0.86)**		**0.46**	**(0.70)**	
	Prop. agree (ppos, pneg)	0.90	(0.56, 0.95)		0.76	(0.62, 0.82)	
	Bias Index, BI	0.03			0.14		
	Prevalence Index, PI	0.78			0.36		
	**Fresh faces and whole liver examination (n = 65)**						
		**cELISA**			**FE**		
		**Positive**	**Negative**	**Total**	**Positive**	**Negative**	**Total**
**Liver exam.**	**Positive**	6 (*8*)	12 (*10*)	18	9 (*11*)	9 (*7*)	18
	**Negative**	2 (*0*)	45 (*47*)	47	2 (*0*)	45 (*47*)	47
	**Total**	8	57	65	11	54	65
	Agreement	6	45	51	9	45	54
	Exp. by chance (nearest whole number)	2	41	43	3	39	42
	Kappa (max attainable)	**0.35**	**(0.54)**		**0.52**	**(0.69)**	
	Prop. agree (ppos, pneg)	0.78	(0.46, 0.87)		0.83	(0.62, 0.89)	
	Bias Index, BI	0.15			0.11		
	Prevalence Index, PI	0.60			0.55		

Maximum attainable kappa is obtained using the italicised numbers in parentheses (i.e., those numbers that would secure maximum agreement between the tests given the (fixed) marginal totals) [28].

### Concurrent diagnoses by FE, cELISA and whole liver examination

Prior to slicing into 1-2cm parallel strips, initial visual inspection revealed obvious scarring in fluke infested tissue of the heaviest burdened livers (i.e., 4 or more fluke) and enabled targeted removal of several specimens. Eighteen of 65 examined livers were found to contain at least one mature (categorised by size; S1 Fig) liver fluke (range: one to 13; only one seemingly immature fluke was found and this was found alongside mature fluke); six were positive by cELISA, and nine were positive by FE (22 positives between the three tests, 14 positives between FE and cELISA, 20 positives between FE and liver, and 20 positives between cELISA and liver) (Table 1; see S3 Table for data stratified by month, sex and estate). Two cELISA positives (both FE negatives) were fluke-free using visual liver inspection, and, two FE positives (both cELISA negatives) were also fluke-free by visual liver inspection (Table 1). Liver examination diagnosed significantly more positives than the cELISA (McNemar’s χ^2^ = 5.79, df = 1, p-value = 0.016), but not more than FE (McNemar’s χ^2^ = 3.27, df = 1, p-value = 0.070)), and differences between prevalence estimated by FE and cELISA were not significant (McNemar’s χ^2^ = 0.44, df = 1, p-value = 0.51).

**Fig 3:**
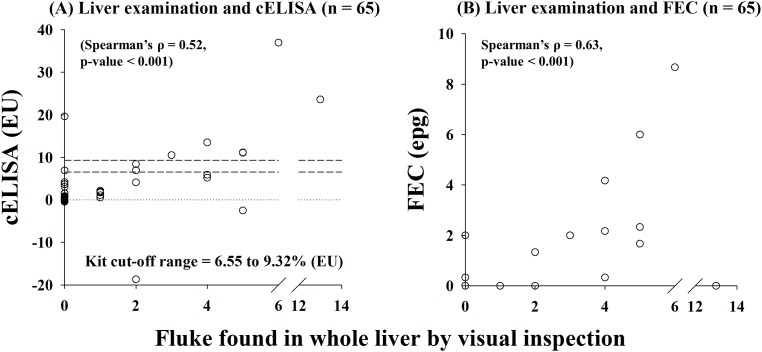
Scatter plots comparing results of FEC and cELISA (BioX, Belgium) diagnostic tests for *F*. *hepatica* infection against concurrent visual inspection of whole livers from red deer for fluke. Faecal samples and livers were collected from carcasses of wild Scottish red deer culled between 2012 and 2014 (n = 65). Livers were sliced and visually inspected for flukes. For the FEC test, results are recorded in eggs per gram of faeces (epg). For the cELISA, results are expressed in ELISA units (EU). Positive diagnosis by the cELISA was recorded for samples where results fell above a cut off derived using a positive reference standard.

At the individual level, agreement between liver examination and cELISA was low (κ_cELISA_ = 0.35), and agreement between liver examination and FE was moderate (κ_FE_ = 0.52) (Table 1); whereas correlation between cELISA (EU) vs. number of fluke (Spearman’s ρ = 0.52, p < 0.001) was similar to the correlation between FEC (epg) vs. number of fluke (Spearman’s ρ = 0.63, p < 0.001) (Fig 3), and the correlation between cELISA and FEC was markedly weaker (Spearman’s ρ = 0.30, p = 0.014; S2 Fig).

Using the 18 known positives classified by liver examination, the sensitivity of the cELISA and FE were 33% (95% CI: 11%–55%) and 50% (95% CI: 32%–78%), respectively; and, there was no significant difference between them (McNemar’s χ^2^ = 0.8, df = 1, p-value = 0.37). Using the 47 apparent negatives, the specificity of the cELISA and the FE were both 96% (95% CI: 90%–100%).

Where whole livers were not available, 52 frozen faecal samples were identified as positive by FE, and 41 of these were positive by cELISA, indicating cELISA sensitivity to patent infection of 79% (95% CI 73%–84%). Similarly, of 18 fresh faecal samples (7 of which did not have an accompanying whole liver) found positive by FE, 9 were positive by cELISA, indicating cELISA sensitivity of 50% (95% CI: 32%–78%).

### Application of the cELISA to quantify *F*. *hepatica* prevalence in Scottish Highland deer

**Fig 4:**
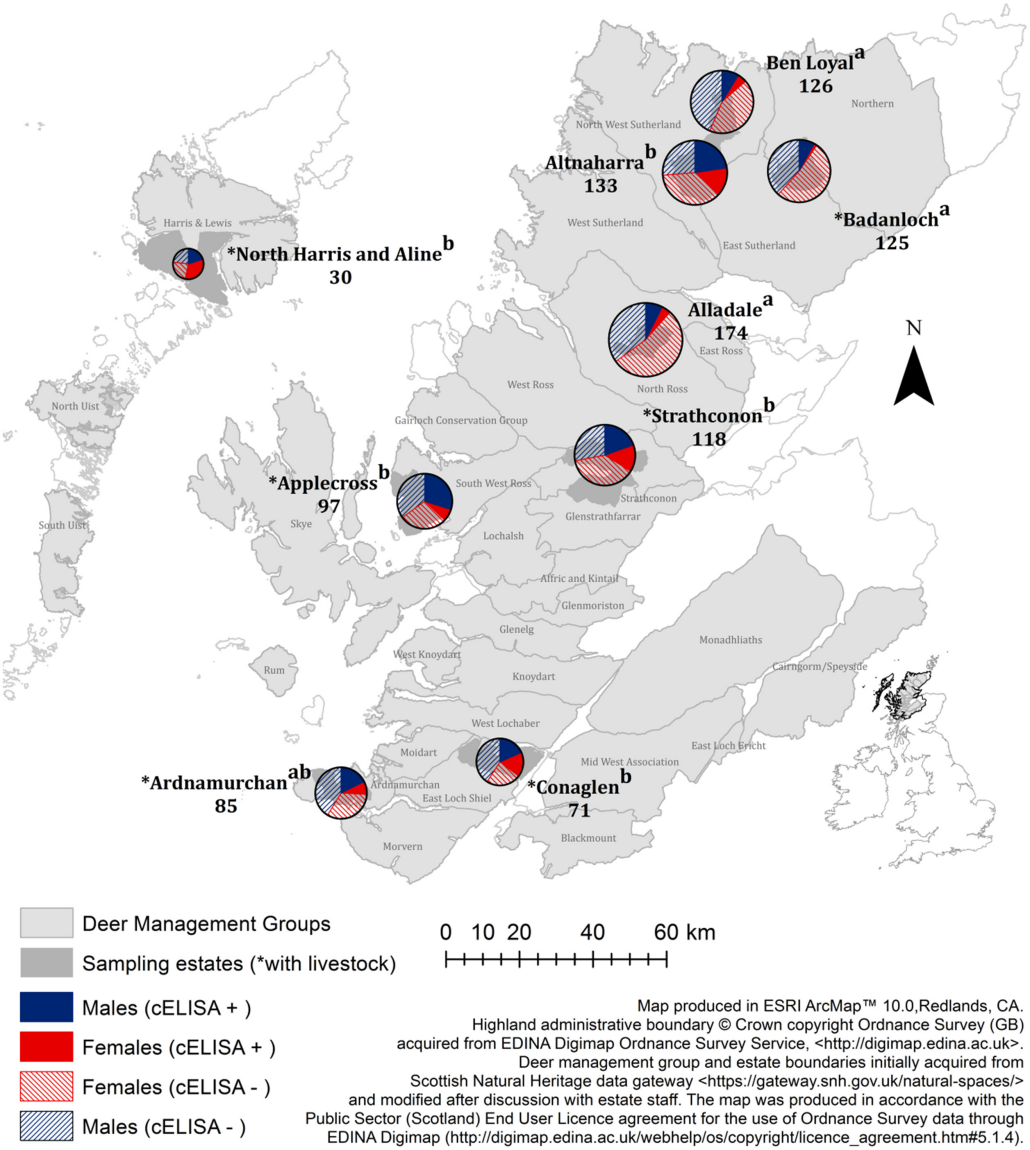
Prevalence of *F*. *hepatica* in red deer in the Scottish Highlands (n = 959). Pie charts illustrate the proportions of infected and non-infected male and female deer in each estate. The size of each pie chart correlates with the number of samples collected on each estate. Overall differences in prevalence between estates are denoted by compact letter descriptors (calculated using Tukey Contrasts in the {glht} function in R), whereby estates that share at least one letter do not have significant differences (at the 5% level) in prevalence. See S4 Table for prevalence stratified by sex and sampling seasons. Livestock presence (*) is noted for estates that contained sheep and/or cattle. All (*) denoted estates had hill sheep, and Applecross, Conaglen and Strathconon also had cattle.

**Fig 5:**
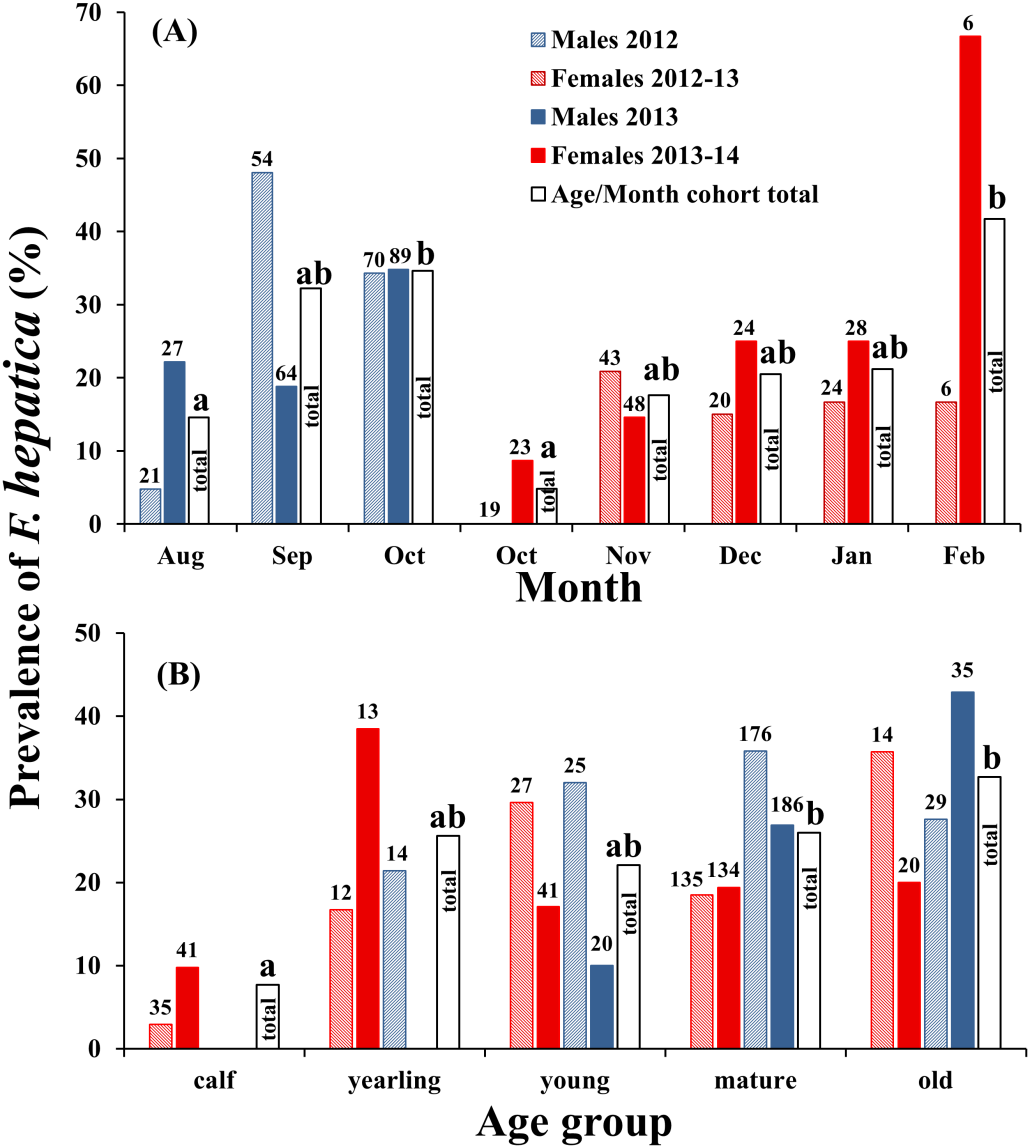
Prevalence of *F*. *hepatica* estimated by cELISA in red deer culled during the 2012–13 and 2013–14 stalking seasons in relation to: (A) Month (mature animals only; n_male_ = 325; n_female_ = 241; n_total_ = 566); and (B) Age group (n_male_ = 485; n_female_ = 472; n_total_ = 957). Significant differences at the 5% level (calculated using Tukey Contrasts in the {glht} function in R) are shown by compact letter descriptors; months/ages sharing a letter did not have significantly different prevalences. For clarity, data for two male calves (one positive in 2012; one negative in 2013) are not illustrated in (B), but were included in the statistical analyses.

Marked geographic differences in *F*. *hepatica* prevalence were found between estates (Fig 4 and S4 Table). Furthermore, overall prevalence in males was greater than in females (male prevalence 30.8%, female prevalence 18.4%; χ^2^ = 19.049, df = 1, p-value < 0.0001), and prevalence of *F*. *hepatica* increased with increasing deer age class and throughout the stalking (sampling) seasons (Fig 5).

## Discussion

This study has demonstrated that the *F*. *hepatica* cELISA is ostensibly more practicable for surveillance in wild red deer than the alternative, FE; and, though cELISA sensitivity to pre-patent infection remains unclear, we have found similar sensitivity to patent infection to that reported in sheep and cattle [17,22], and negligible uncertainty surrounding its well-recognised high specificity [17,31]. Our data has further revealed marked geographic, temporal and age related variation in the prevalence of *F*. *hepatica* in wild red deer in the Scottish Highlands.

### Diagnostic evaluation

Practicality of sample handling is a key consideration when gathering wildlife surveillance data; hence, freezing faecal samples was our chief approach because it was straightforward for gamekeepers in remote areas of the Highlands to collect and store deer faeces. However, the standard diagnostic for *F*. *hepatica* in deer, the FE, is intended for fresh faeces; thus, fresh samples were collected during 2013–14 for comparison. Freezing of faeces destined for FE is advised against because it significantly reduces egg detectability for nematodes such as *Haemonchus contortus* in sheep [32] and in deer [33], though it does not necessarily reduce detectability for all trematodes (e.g., *Dicrocoelium dendriticum* in sheep [34]). Here, we were able to identify recognisable *F*. *hepatica* eggs in frozen samples (S3 Fig as an example), though egg collapse (and resultant reduction in detection) may well have contributed to disparities in prevalence estimates between cELISA and FE diagnoses (S1 and S2 Tables). In addition, *F*. *hepatica* excretory-secretory antigens (when frozen on the day of collection) appear to remain stable for at least a year in frozen red deer faeces, as reported (though for a shorter timespan) in domestic ruminants [18,19]; however, it is unclear for how long antigens remain stable in fresh samples. Owing to our sampling design, up to seven days post-sampling/pre-shipping delay (for fresh samples) left potential for antigen degradation—perhaps reducing detectability of already low burdens (Fig 3), as reported in human faeces [35].

To assess the agreement between the cELISA and the FE, we examined individual level paired diagnoses, and population level prevalence estimates. At the individual level, we noted a high proportion of agreement between the cELISA and FE, but the low underlying prevalence in fresh samples reduced the potential for agreement beyond chance, resulting in moderate kappa (Table 1). In frozen samples, underlying prevalence was greater, indicating that the moderate agreement between the cELISA and FE was more likely a consequence of true disparities in diagnoses—perhaps caused by egg collapse. To our knowledge, agreement (estimated by kappa) has not been reported in the peer-reviewed literature for the *F*. *hepatica* cELISA and FE, and instead diagnostic performance is compared in terms of population prevalence; e.g., percent positive samples based on FE, serum-antibody and copro-antigen ELISAs [36]. Nevertheless, we can refer to publically available data for FE and cELISA in sheep naturally exposed to *F*. *hepatica* metacercariae [37]. These data show stronger agreement (κ = 0.83) along with generally higher egg counts (mean 12.5 epg) than observed in our deer study. Moreover, given the median FEC that corresponded with positive cELISA diagnoses in the sheep was 11.8 epg, strong agreement is not surprising. In contrast, we observed excess of 6 epg in only five of 146 fresh and two of 207 frozen deer faecal samples, likely reducing the potential for concurrent positive cELISA diagnoses (at least in fresh samples; Fig 2).

In terms of population level agreement, cELISA and FE prevalence were strongly correlated (Fig 1), but the cELISA regularly diagnosed greater prevalence than the FE, which (assuming both tests are highly specific) alluded to greater sensitivity of the cELISA. To further explore these diagnostic parameters, we estimated sensitivity and specificity of both tests against our liver examination reference standard (limited to fresh faecal samples); and, indeed, the specificity of both methods was very high, but there was no difference in sensitivity.

In frozen samples, we had no concurrent liver reference standard against which to estimate cELISA and FE sensitivity and specificity; nevertheless, as we were concerned primarily with the suitability of the cELISA for surveillance, its sensitivity based on patent infection was similar to that in sheep and cattle with 1–2 fluke [17,22], underlining its suitability to wild deer surveillance based on frozen samples. If indeed an improvement on this sensitivity were desired, it may be worth investigating the use of a customised cut-off of the cELISA (though it would be challenging at best to obtain known non-infected wild red deer), or (more realistically) modifying laboratory procedures (e.g., increasing sample incubation time), or both [18,22].

Finally, in terms of diagnostic evaluation, we considered latent class models (LCM) as described by Hui and Walter [38], but our data did not meet the assumption of independent errors; i.e., the FE, liver examination and cELISA all work on the principal of detecting parasites, thus the higher the fluke burden, the more detectable they become in faeces (higher antigen concentrations, more eggs [36]) and livers (Figs 2 and 3). An alternative approach of Bayesian LCM (where the independent errors assumption may be relaxed [39]) was also considered, but was deemed inappropriate because we had insufficient knowledge of FE or liver examination sensitivity or underlying population prevalence in the Highland deer population; hence, we could not specify enough parameter distributions (independent of our data) a priori, which is required for such an approach. Lastly, we note that the cELISA is reported to detect immature fluke [17] (pre-patent infection) (though this has not been demonstrated in the field [20]), whereas FE cannot. If positive cELISA diagnoses in deer are indeed attributed to pre-patent infection, then the diagnostics are not designed to detect the same disease state, which itself is incompatible with the assumption specified by Hui and Walter [38]—that the tests should be conditionally independent given *the* true (but latent) disease state.

We note some peculiarities of our results and potential limitations of the diagnostics. Firstly, the two cELISA positives (both FE negatives) that were fluke-free by visual liver inspection and two FE positives (both cELISA negatives) also fluke-free by visual liver inspection are potentially a consequence of imperfect visual liver inspection, or, loss of fluke during larder collection of whole livers (Table 1 and Fig 3). Indeed, fluke can be lost in sheep at post-mortem as a consequence of removal of the gall bladder (which provides a physical obstacle to escaping fluke); red deer on the other hand do not have a gall bladder, so removal of whole liver may lead to loss of mature fluke directly out of the large bile duct. Alternatively, the FE disparities could be explained by egg ingestion and subsequent passage in the faeces of non-infected deer—it is worth noting that these two FE positives (liver examination negatives) were females, and female herds typically “heft” to the same areas in which they are born—perhaps making them more likely to ingest eggs. In addition, there is potential for misidentification of rumen fluke (*Calicophoron daubneyi*) eggs as *F*. *hepatica* eggs, though rumen fluke infection in wild deer has only recently been reported in the Republic of Ireland [4], and there have been no reported cases in wild Scottish red deer. In terms of the cELISA, the positive value that falls just above the 6.55% (EU) cut-off line (where no flukes were found) could be a consequence of the (unknown) variation of cELISA titres for samples collected from uninfected red deer; i.e., it may be a false positive. This may be a consequence of the manufacturer’s cut-off value, which is calculated as a percentage of the positive reference antigen supplied with the kit, so does not take titre variation of truly negative samples into account. The positive cELISA titre that falls at 20 EU (where no flukes were found) is perhaps a consequence of cross-contamination during sample handling and/or cross-reactivity with other molecules or antigens present in faeces, though this has not been observed where deliberately explored with other fluke species; e.g., *Dicrocoelium dentriticum* and *Paramphistomum cervi* [17], and Calicophoron *daubneyi* [31].

A peculiarity of *F*. *hepatica* in deer, which may explain the relatively low sensitivities of cELISA and FE to the observed burdens, relates to the way in which fluke inhabit deer livers. Here, it was not clear whether the “pockets” containing fluke ran unobstructed to the bile ducts; if obstructed, there would be an incomplete path for eggs and antigens to enter the digestive system, which would impair detection. Finally, red deer have no gall bladder (unlike sheep, for which the kit is designed), so eggs and antigens theoretically form a constant trickle into the digestive system and do not pool in an intermittently evacuated reservoir from which antigen concentration may (in sheep) be amplified; thus, only infection with burdens in excess of six fluke may reliably detected by cELISA.

### *F*. *hepatica* prevalence in wild Scottish red deer

To our knowledge, this is the first Scottish Highland regional survey of *F*. *hepatica* in wild red deer, though our estimates of prevalence fall with the range of sub-regional European studies; e.g., in Spain (34% by serum ELISA and 7% at necropsy) [2] and in Belarus (33% at necropsy) [5].

Greater *F*. *hepatica* prevalence was observed in males than in female; reflecting male-biased parasitism observed in other ungulates (e.g., gastrointestinal worm burden attributed to stress hormones in chamois (*Rupicapra rupicapra rupicapra*) [40], and rumen fluke prevalence in cattle [41]). We eliminated the potentially confounding factor of age by considering only mature animals, though the observed sex bias remains entangled with temporal effects, as males are harvested earlier (August—October) than females (October—February). We speculate that males in our study would have had higher daily food intake owing to the disparity in size between the sexes; hence, males would have a higher probability of eating infective metacercariae than females.

Prevalence of *F*. *hepatica* also increased as both male and female stalking seasons progressed (Fig 5). Such a temporal signal is inherently related to the parasite life cycle, but it is difficult to disentangle this signal from the unknown longevity of *F*. *hepatica* in red deer. With this in mind we looked for an overall age-related increase in prevalence and a temporal increase in prevalence in mature deer only. Interestingly, the age related increase in prevalence was not significant between yearling, young, mature and old animals (Fig 5), perhaps suggesting that the longevity of fluke in deer may not be similar to sheep (5 + years) [42] and goats (11 + years) [43]; indeed, the marked increase in prevalence between the beginning and end of both the male and female stalking seasons suggests that fluke prevalence is indicative of annual reinfection and points to a perhaps shorter and more similar longevity to that in cattle (26 + months) [44]. The liver tissue response in red deer associated with *F*. *hepatica* has not been extensively documented; however, where reported, it is characterised by thickened cyst (i.e., “pockets” as observed in our study) walls, though there is not the calcification that is typically observed in cattle [45]. Further complicating matters, if fluke survive for more than a year inside the final deer host, and considering that wild red deer, unlike livestock, are not treated with flukicides, infections detected early in the stalking season could represent persistent infections from the previous calendar years, or new infection sustained in the current calendar year.

This study is the first to quantify the extent of geographic variation in *F*. *hepatica* prevalence in wild deer in the Scottish Highland region. Here, we have observed low *F*. *hepatica* prevalence where livestock are not present and high prevalence in the presence of hill sheep (and three estates with small numbers of cattle) (Fig 4). Interestingly, there are two notable exceptions to this: i) low prevalence was observed at Badanloch, where there is an extensive hill sheep farm, and ii) high prevalence was observed in Altnaharra, where no livestock were present. In addition, the marked disparity in prevalence between Altnaharra and its neighbouring estate, Ben Loyal, reflects the sub-regional (post code scale) variation in prevalence that has been observed in livestock in England and Wales [46]. With these observations in mind, sub-estate scale (i.e., deer home-range) environmental variation associated with probability of infection remains to be explored.

## Conclusions

We have highlighted two advantages of cELISA over FE in terms of *F*. *hepatica* surveillance in red deer in a remote region: i) owing to its propensity to diagnose greater prevalence than FE in frozen sample cohorts (and its apparent high specificity), the cELISA has seemingly greater sensitivity; and ii) reduced labour and the ability to process large batches. We acknowledge that the lack of true (known) negative individuals studied here (typical of a study involving wild populations) and the lack of a gold-standard diagnostic test made traditional evaluation of the cELISA’s sensitivity and specificity imperfect, but, emphasise that even our conservative estimates of test parameters support the use of the cELISA for wild red deer surveillance. For completeness, a larger, focussed evaluation of cELISA sensitivity in red deer is desirable. In the meantime, our observation of geographic variation in particular highlights that further research into *F*. *hepatica* and the factors associated with wild deer infection is warranted. We advocate further application of cELISA to wild red deer, and are intrigued as to its potential application to other potentially important wild liver fluke hosts such as other cervids or even leporids.

## Supporting Information

S1 Fig Liver fluke specimens from an Altnaharra female. Note that based on size (scale centimetres), these fluke were considered mature, and therefore evidence of patent infection. Where fluke segments were found during liver examination, only heads were counted; identified by the presence of ventral suckers as highlighted.
(TIF)

S2 Fig Scatter plots comparing results of FEC and cELISA (BioX, Belgium) diagnostic tests for *F*. *hepatica* infection (where concurrent diagnoses by liver examination were also undertaken; n = 65). Samples and livers were collected from carcasses of wild Scottish red deer culled between 2012 and 2014. For the FEC test, results are recorded in eggs per gram of faeces (epg). For the cELISA, results are expressed in ELISA units (EU). Positive diagnosis by the cELISA was recorded for samples where results fell above a cut off derived using a positive reference standard.
(TIF)

S3 Fig A *F*. *hepatica* egg (typically 130–145μm in length) from a frozen faecal sample on a contact plate viewed under a dissecting microscope. The other faecal matter visible in this image is counter-stained with 1% methylene blue.
(TIF)

S1 Table *F*. *hepatica* prevalence estimated by FE and cELISA, in relation to sex, year and month. Fresh samples in bold.
(DOCX)

S2 Table *F*. *hepatica* prevalence estimated by FEC and cELISA, in relation to sex, year and estate. Fresh samples are highlighted in bold.
(DOCX)

S3 Table *F*. *hepatica* prevalence estimated by FEC, cELISA and liver examination, in relation to sex, month and estate. All faecal samples were stored fresh. Significant differences (identified using Chi-square test of independence and further explored using Tukey contrasts) are indicated by compact letter descriptors; diagnostic methods sharing a letter were not significantly different from each other.
(DOCX)

S4 Table *F*. *hepatica* prevalence estimated by cELISA in relation to sex and estate. Significant differences (at the 5% significance level, identified using Chi-square test of independence and further explored using Tukey contrasts) between estates are indicated by compact letter descriptors, whereby estates that share a letter did not have significantly different prevalences.
(DOCX)

S1 Text Example of cross tabulation comparison between two diagnostic methods. An explanation of the kappa statistic and its associated equations and interpretational parameters.
(DOCX)
