# Supplementary figures and images for: Prevalence of Liver Fluke (Fasciola hepatica) in Wild Red Deer (Cervus elaphus): Coproantigen ELISA Is a Practicable Alternative to Faecal Egg Counting for Surveillance in Remote Populations
Source: PLoS One. 2016 Sep 6;11(9):e0162420. doi: 10.1371/journal.pone.0162420 (PMC5012657; doi:10.1371/journal.pone.0162420)

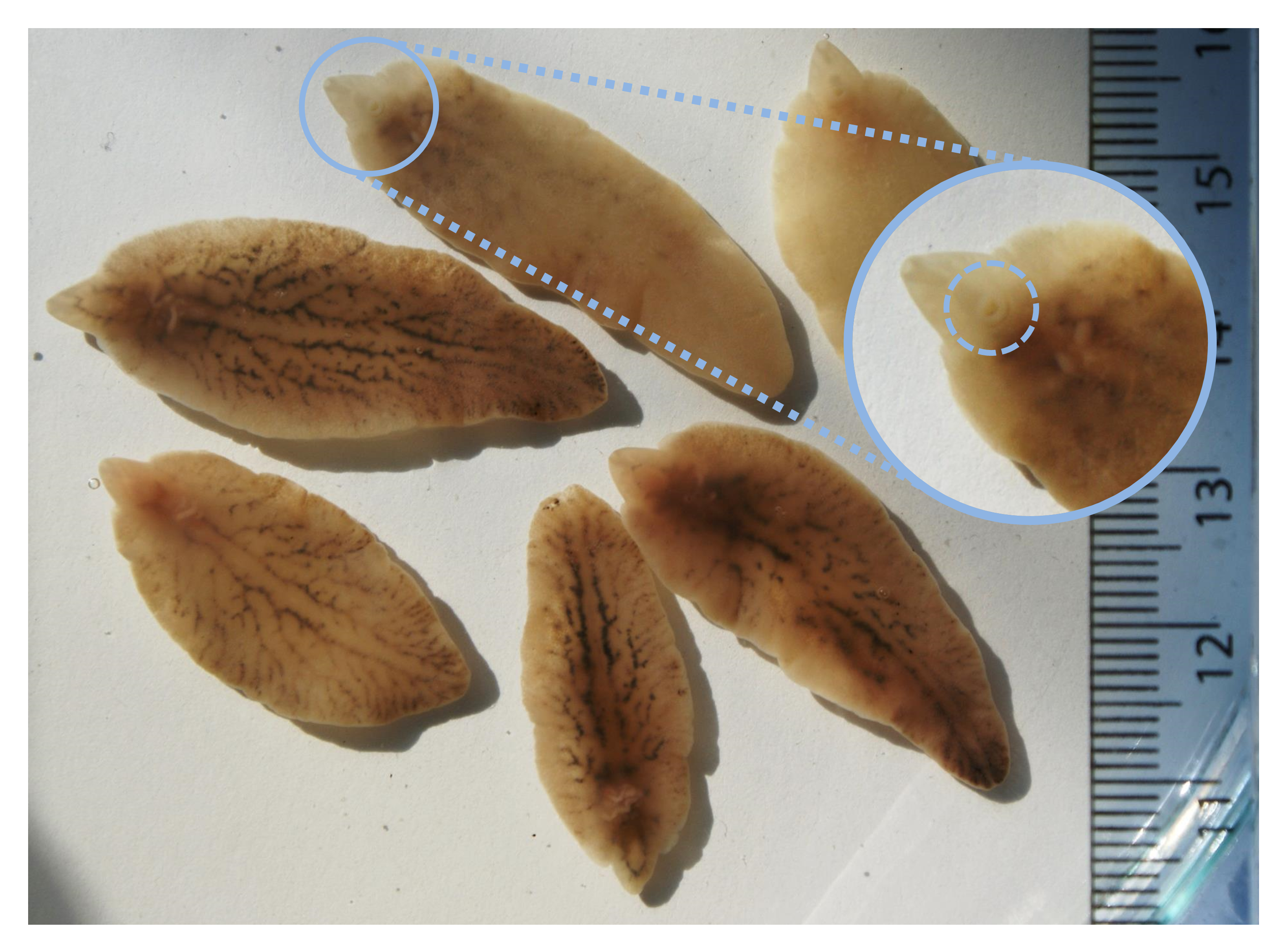

Supplement: S1 Fig — Note that based on size (scale centimetres), these fluke were considered mature, and therefore evidence of patent infection. Where fluke segments were found during liver examination, only heads were counted; identified by the presence of ventral suckers as highlighted. (TIF) [file pone.0162420.s001.tif]

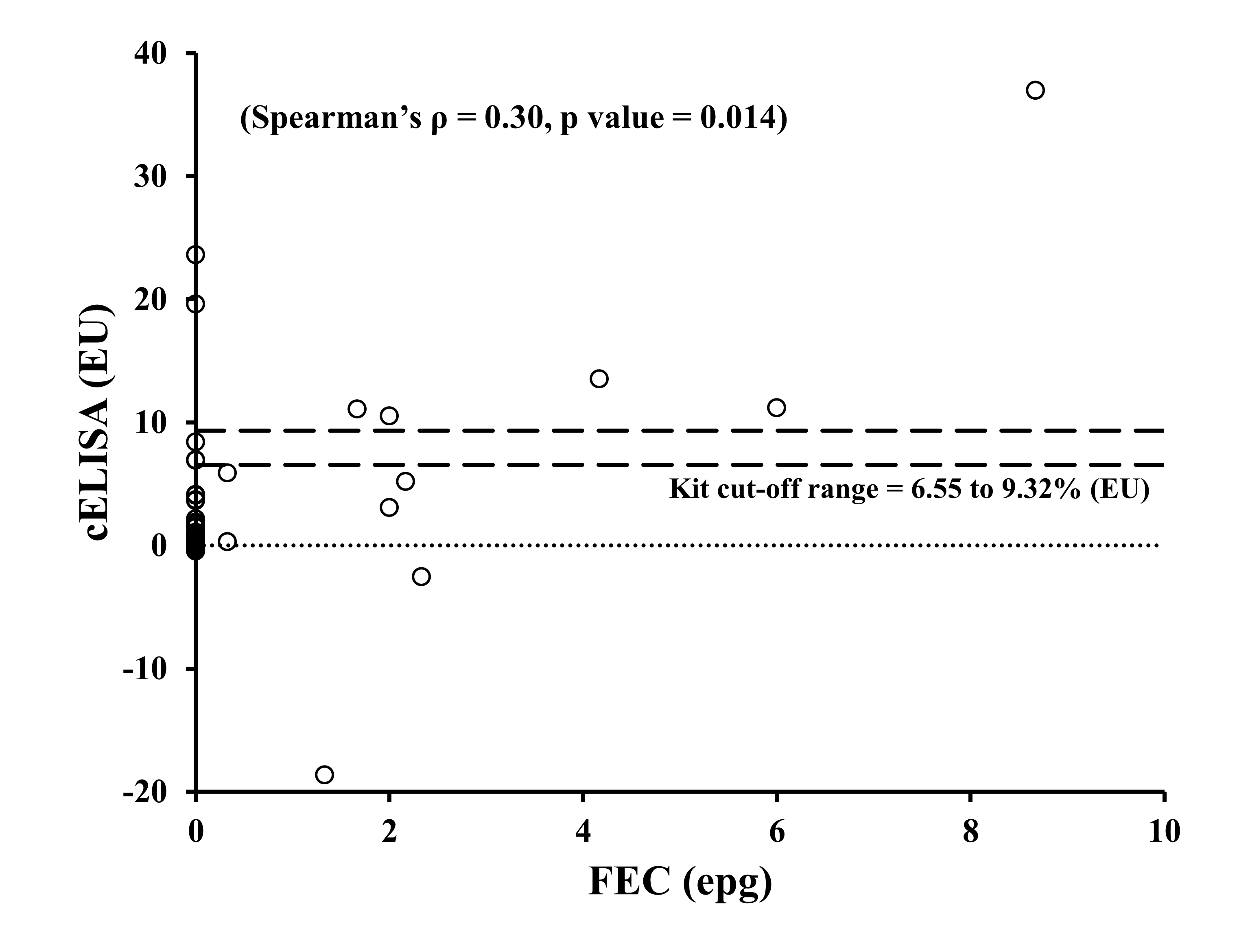

Supplement: S2 Fig — Samples and livers were collected from carcasses of wild Scottish red deer culled between 2012 and 2014. For the FEC test, results are recorded in eggs per gram of faeces (epg). For the cELISA, results are expressed in ELISA units (EU). Positive diagnosis by the cELISA was recorded for samples where results fell above a cut off derived using a positive reference standard. (TIF) [file pone.0162420.s002.tif]

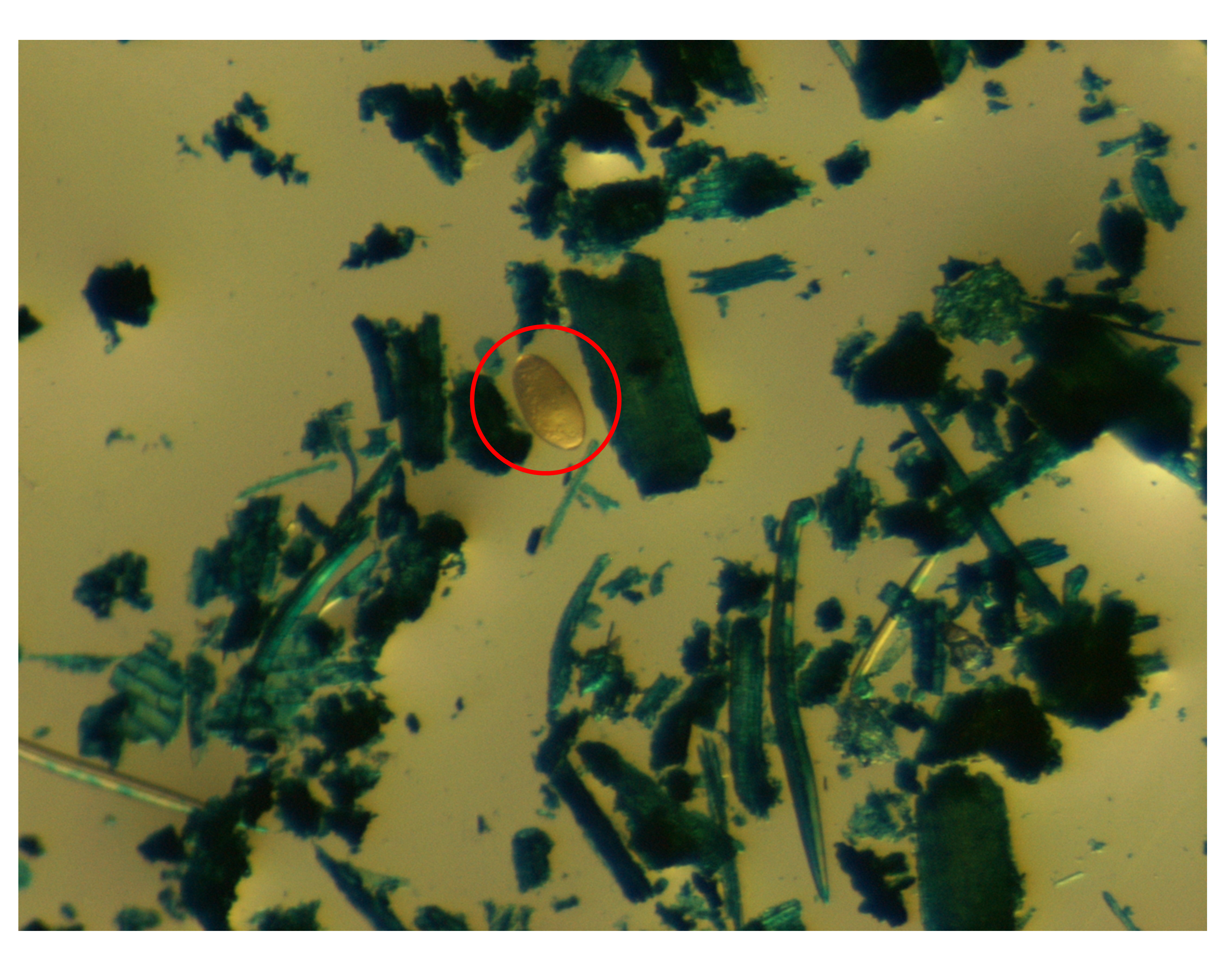

Supplement: S3 Fig — The other faecal matter visible in this image is counter-stained with 1% methylene blue. (TIF) [file pone.0162420.s003.tif]
