# Supplementary material for: Prevalence of Liver Fluke (Fasciola hepatica) in Wild Red Deer (Cervus elaphus): Coproantigen ELISA Is a Practicable Alternative to Faecal Egg Counting for Surveillance in Remote Populations
Source: PLoS One. 2016 Sep 6;11(9):e0162420. doi: 10.1371/journal.pone.0162420 (PMC5012657; doi:10.1371/journal.pone.0162420)
Supplement: S1 Table — Fresh samples in bold. (DOCX) [file pone.0162420.s004.docx]

**Table S1. *F. hepatica* prevalence estimated by FE and cELISA, in relation to sex, year and month.** Fresh samples in bold.

|  |  |  |  | **YEAR** | | | | | | | | | | | | | | | | | | | | |
| --- | --- | --- | --- | --- | --- | --- | --- | --- | --- | --- | --- | --- | --- | --- | --- | --- | --- | --- | --- | --- | --- | --- | --- | --- |
|  |  |  |  | **2012-13** | | |  |  |  | **2013-14** | | | | | | | | |  |  |  | **2012-14** | | |
|  |  |  |  | **Frozen** | | |  |  |  | **Frozen** | | |  |  |  | **Fresh** | | |  |  |  | **Frozen** | | |
| sex | | |  | Male | Female | Overall  (***) |  |  |  | Male NS | female | Overall (NS) |  |  |  | **male** | **female** | **Overall (NS)** |  |  |  | Male | Female | Overall (***) |
| n | | |  | 86 | 73 | 159 |  |  |  | 29 | 19 | 48 |  |  |  | **59** | **87** | **146** |  |  |  | 115 | 92 | 207 |
| FEC | | |  | 32.6 | 17.8 | 25.8 |  |  |  | 31.0 | 10.5 | 22.9 |  |  |  | **11.9** | **12.6** | **12.3** |  |  |  | 31.6 | 16.3 | 25.1 |
| cELISA | | |  | 54.7 | 28.8 | 42.8 |  |  |  | 34.5 | 10.5 | 25.0 |  |  |  | **11.9** | **8.0** | **9.6** |  |  |  | 48.7 | 25.0 | 38.6 |
| **MONTH** | | | | | | | | | | | | | | | | | | | | | | | | |
|  |  | **Aug** | | | | |  | **Sep** | | | | |  | **Oct (Male)** | | | | |  | **Oct (Female)** | | | | |
|  |  | 2012 |  | 2013 | | |  | 2012 |  | 2013 | | |  | 2012 |  | 2013 | | |  | 2012 |  | 2013 | | |
| storage |  | **frozen** |  | **frozen** | **fresh** | overall |  | **frozen** |  | **frozen** | **fresh** | overall |  | **frozen** |  | **frozen** | **fresh** | overall |  | **frozen** |  | **frozen** | **fresh** | overall |
| n |  | 18 |  | 4 | **15** | 19 |  | 25 |  | 2 | **29** | 31 |  | 31 |  | 22 | **15** | 37 |  | 6 |  | 1 | **20** | 21 |
| FEC |  | 16.7 |  | 0.0 | **20.0** | 15.8 |  | 32.0 |  | 50.0 | **0.0** | 3.2 |  | 41.9 |  | 36.4 | **26.7** | 32.4 |  | 16.7 |  | 100.0 | **0.0** | 4.8 |
| cELISA |  | 22.2 |  | 50.0 | **6.7** | 15.8 |  | 80.0 |  | 0.0 | **10.3** | 9.7 |  | 51.6 |  | 36.4 | **20.0** | 29.7 |  | 0.0 |  | 100.0 | **0.0** | 4.8 |
|  |  | **Nov** | | | | |  | **Dec** | | | | |  | **Jan** | | | | |  | **Feb** | | | | |
|  |  | 2012 |  | 2013 | | |  | 2012 |  | 2013 | | |  | 2013 |  | 2014 | | |  | 2013 |  | 2014 | | |
| storage |  | **frozen** |  | **frozen** | **fresh** | overall |  | **frozen** |  | **frozen** | **fresh** | overall |  | **frozen** |  | **frozen** | **fresh** | overall |  | **frozen** |  | **frozen** | **fresh** | overall |
| n |  | 29 |  | 3 | **39** | 42 |  | 12 |  | 3 | **13** | 16 |  | 4 |  | 8 | **13** | 21 |  | 3 |  | 4 | **2** | 6 |
| FEC |  | 6.9 |  | 33.3 | **17.9** | 19.0 |  | 33.3 |  | 0.0 | **23.1** | 18.8 |  | 0.0 |  | 0.0 | **7.7** | 4.8 |  | 0.0 |  | 0.0 | **0.0** | 0.0 |
| cELISA |  | 17.2 |  | 0.0 | **10.3** | 9.5 |  | 33.3 |  | 0.0 | **15.4** | 12.5 |  | 25.0 |  | 0.0 | **7.7** | 4.8 |  | 66.7 |  | 25.0 | **0.0** | 16.7 |

Diagnoses in fresh samples are highlighted in bold. Significant differences at the 5% level between prevalences (calculated by McNemar’s χ^2^) estimated by FE and cELISA are denoted by symbols: p-value > 0.05 (NS), < 0.001 (***).).
