# Supplementary material for: Prevalence of Liver Fluke (Fasciola hepatica) in Wild Red Deer (Cervus elaphus): Coproantigen ELISA Is a Practicable Alternative to Faecal Egg Counting for Surveillance in Remote Populations
Source: PLoS One. 2016 Sep 6;11(9):e0162420. doi: 10.1371/journal.pone.0162420 (PMC5012657; doi:10.1371/journal.pone.0162420)
Supplement: S2 Table — Fresh samples are highlighted in bold. (DOCX) [file pone.0162420.s005.docx]

**Table S2. *F. hepatica* prevalence estimated by FEC and cELISA, in relation to sex, year and estate. Fresh sample are highlighted in bold.**

|  |  | **ESTATE** | | | | | | | | | | | | | | | | | | | | | | |
| --- | --- | --- | --- | --- | --- | --- | --- | --- | --- | --- | --- | --- | --- | --- | --- | --- | --- | --- | --- | --- | --- | --- | --- | --- |
|  |  | **Altnaharra** | | | | | | |  | **Badanloch** | | | | | | |  | **Ben Loyal** | | | | | | |
|  |  | Year | | | | | | |  | Year | | | | | | |  | Year | | | | | | |
|  |  | 2012-13 | | |  | 2013-14 | | |  | 2012-13 | | |  | 2013-14 | | |  | 2012-13 | | |  | 2013-14 | | |
|  |  | **Frozen** | | |  | Frozen | **Fresh** |  |  | **Frozen** | | |  | **Fresh** | | |  | **Frozen** | | |  | **Fresh** | | |
| sex |  | male | female | overall |  | male | **female** | overall |  | male | female | overall |  | **male** | **female** | **overall** |  | male | female | overall |  | **male** | **female** | **overall** |
| n |  | 14 | 13 | 27 |  | 9 | **32** | 41 |  | 4 | 10 | 14 |  | **26** | **35** | **61** |  | 14 | 6 | 20 |  | **33** | **20** | **53** |
| FEC |  | 50.0 | 30.8 | 40.7 |  | 33.3 | **31.3** | 31.7 |  | 0.0 | 10.0 | 7.1 |  | **11.5** | **0.0** | **4.9** |  | 14.3 | 0.0 | 10.0 |  | **25.0** | **5.0** | **9.4** |
| cELISA |  | 57.1 | 53.8 | 55.6 |  | 55.6 | **15.6** | 24.4 |  | 50.0 | 0.0 | 14.3 |  | **15.4** | **2.9** | **8.2** |  | 28.6 | 33.3 | 30.0 |  | **18.8** | **5.0** | **7.5** |
|  |  |  |  |  |  |  |  |  |  |  |  |  |  |  |  |  |  |  |  |  |  |  |  |  |
|  |  | **Alladale** | | | | | | |  | **Applecross** | | | | | | |  | **Strathconon** | | | | | | |
|  |  | Year | | | | | | |  | Year | | | | | | |  | Year | | | | | | |
|  |  | 2012-13 | | |  | 2013-14 | | |  | 2012-13 | | |  | 2013-14 | | |  | 2012-13 | | |  | 2013-14 | | |
|  |  | **Frozen** | | |  | **Frozen** | | |  | **Frozen** | | |  | **Frozen** | | |  | **Frozen** | | |  | **Frozen** | | |
| sex |  | male | female | overall |  | male | female | overall |  | male | female | overall |  | male | female | overall |  | male | female | overall |  | male | female | overall |
| n |  | 10 | 18 | 28 |  | 11 | 3 | 14 |  | 15 | 9 | 24 |  | 6 | 10 | 16 |  | 13 | 11 | 24 |  | 3 | 6 | 9 |
| FEC |  | 7.1 | 0.0 | 3.6 |  | 18.2 | 66.7 | 28.6 |  | 60.0 | 33.3 | 50.0 |  | 66.7 | 0.0 | 25.0 |  | 38.5 | 45.5 | 41.7 |  | 0.0 | 0.0 | 0.0 |
| cELISA |  | 14.3 | 14.3 | 17.9 |  | 9.1 | 33.3 | 14.3 |  | 73.3 | 22.2 | 54.2 |  | 33.3 | 10.0 | 18.8 |  | 53.8 | 45.5 | 50.0 |  | 66.7 | 0.0 | 22.2 |
|  |  |  |  |  |  |  |  |  |  |  |  |  |  |  |  |  |  |  |  |  |  |  |  |  |
|  |  | **Conaglen** | | |  | **North Harris and Aline** | | |  | **Ardnamurchan** | | |  |  |  |  |  |  |  |  |  |  |  |  |
|  |  | Year | | |  | Year | | |  | Year | | |  |  |  |  |  |  |  |  |  |  |  |  |
|  |  | 2012-13 | | |  | 2012-13 | | |  | 2012-13 | | |  |  |  |  |  |  |  |  |  |  |  |  |
|  |  | **Frozen** | | |  | **Frozen** | | |  | **Frozen** | | |  |  |  |  |  |  |  |  |  |  |  |  |
| sex |  | male | female | overall |  | male | female | overall |  | male | female | overall |  |  |  |  |  |  |  |  |  |  |  |  |
| n |  | 0 | 3 | 3 |  | 10 | 1 | 11 |  | 6 | 2 | 8 |  |  |  |  |  |  |  |  |  |  |  |  |
| FEC |  | NA | 0.0 | 0.0 |  | 40.0 | 0.0 | 36.4 |  | 0.0 | 0.0 | 0.0 |  |  |  |  |  |  |  |  |  |  |  |  |
| cELISA |  | NA | 66.7 | 66.7 |  | 60.0 | 100.0 | 63.6 |  | 100.0 | 0.0 | 75.0 |  |  |  |  |  |  |  |  |  |  |  |  |
