# Supplementary material for: Prevalence of Liver Fluke (Fasciola hepatica) in Wild Red Deer (Cervus elaphus): Coproantigen ELISA Is a Practicable Alternative to Faecal Egg Counting for Surveillance in Remote Populations
Source: PLoS One. 2016 Sep 6;11(9):e0162420. doi: 10.1371/journal.pone.0162420 (PMC5012657; doi:10.1371/journal.pone.0162420)
Supplement: S3 Table — Significant differences (identified using Chi-square test of independence and further explored using Tukey contrasts) are indicated by compact letter descriptors; diagnostic methods sharing a letter were not significantly different from each other. (DOCX) [file pone.0162420.s006.docx]

**Table S3. *F. hepatica* prevalence estimated by FEC, cELISA and liver examination, in relation to sex, month and estate.** All faecal samples were stored fresh. Significant differences (calculated using McNemar’s test) are indicated by compact letter descriptors; diagnostic methods sharing a letter were not significantly different from each other.

|  |  |  | **MONTH** | | | | | | | | |  |  |  |  |  |  |
| --- | --- | --- | --- | --- | --- | --- | --- | --- | --- | --- | --- | --- | --- | --- | --- | --- | --- |
|  |  |  | Male | | |  | Female | | | | |  | **Totals** | | | | |
| **ESTATE** | method |  | **Aug** |  | **Sep** |  | **Oct** |  | **Nov** |  | **Dec** |  | **males** |  | **females** |  | **both sexes** |
| **Altnaharra** | **FEC** |  |  |  |  |  |  |  | 28.0 |  | 42.9 |  |  |  | 31.3 |  |  |
|  | **cELISA** |  |  |  |  |  |  |  | 12.0 |  | 28.6 |  |  |  | 15.6 |  |  |
|  | **liver** |  |  |  |  |  |  |  | 32.0 |  | 42.9 |  |  |  | 34.4 |  |  |
|  | **n** |  |  |  |  |  |  |  | 25 |  | 7 |  |  |  | 32 |  |  |
| **Badanloch** | **FEC** |  | 50.0 |  | 0.0 |  | 0.0 |  | 0.0 |  |  |  | 6.3 |  | 0.0 |  | 3.0 |
|  | **cELISA** |  | 0.0 |  | 21.4 |  | 0.0 |  | 0.0 |  |  |  | 18.8 |  | 0.0 |  | 9.1 |
|  | **liver** |  | 50.0 |  | 14.3 |  | 26.7 |  | 0.0 |  |  |  | 18.8 |  | 23.5 |  | 21.2 |
|  | **n** |  | 2 |  | 14 |  | 15 |  | 2 |  |  |  | 16 |  | 17 |  | 33 |
| **Totals** | **FEC** |  | 50.0 |  | 0.0 |  | 0.0 |  | 25.9 |  | 42.9 |  | 6.3 |  | 20.4 |  | 16.9^ab^ |
|  | **cELISA** |  | 0.0 |  | 21.4 |  | 0.0 |  | 11.1 |  | 28.6 |  | 18.8 |  | 10.2 |  | 12.3^a^ |
|  | **liver** |  | 50.0 |  | 14.3 |  | 26.7 |  | 29.6 |  | 42.9 |  | 18.8 |  | 30.6 |  | 27.7^b^ |
|  | **n** |  | 2 |  | 14 |  | 15 |  | 27 |  | 7 |  | 16 |  | 49 |  | 65 |
