# Supplementary material for: Prevalence of Liver Fluke (Fasciola hepatica) in Wild Red Deer (Cervus elaphus): Coproantigen ELISA Is a Practicable Alternative to Faecal Egg Counting for Surveillance in Remote Populations
Source: PLoS One. 2016 Sep 6;11(9):e0162420. doi: 10.1371/journal.pone.0162420 (PMC5012657; doi:10.1371/journal.pone.0162420)
Supplement: S4 Table — Significant differences (at the 5% significance level, identified using Chi-square test of independence and further explored using Tukey contrasts) between estates are indicated by compact letter descriptors, whereby estates that share a letter did not have significantly different prevalences. (DOCX) [file pone.0162420.s007.docx]

**Table S4. *F. hepatica* prevalence estimated by cELISA in relation to sex and estate.** Significant differences (at the 5% significance level, calculated using Chi-square test of independence and Tukey Contrasts) are indicated by compact letter descriptors, whereby estates that share a letter did not have significantly different prevalences.

|  | **2012-13** | | | | | | | |
| --- | --- | --- | --- | --- | --- | --- | --- | --- |
|  | **male** | |  | **female** | |  | **overall** | |
| **Estate** | **n** | **prevalence** |  | **n** | **prevalence** |  | **n** | **prevalence** |
| Alladale | 33 | 24.2a |  | 48 | 8.3a |  | 81 | 14.8a |
| Altnaharra | 33 | 42.4a |  | 34 | 38.2b |  | 67 | 40.3b |
| Applecross | 34 | 44.1a |  | 15 | 13.3ab |  | 49 | 34.7ab |
| Ardnamurchan | 21 | 38.1a |  | 10 | 10.0ab |  | 31 | 29ab |
| Badanloch | 31 | 19.4a |  | 32 | 3.1ab |  | 63 | 11.1a |
| Ben Loyal | 33 | 24.2a |  | 39 | 10.3ab |  | 72 | 16.7ab |
| Conaglen | 21 | 33.3a |  | 11 | 45.5ab |  | 32 | 37.5ab |
| NHT and Aline | 11 | 54.5a |  | 1 | 100.0ab |  | 12 | 58.3b |
| Strathconon | 28 | 39.3a |  | 33 | 30.3ab |  | 61 | 34.4ab |
| All | 245 | 33.9 |  | 223 | 18.4 |  | 468 | 26.5 |
|  | **2013-14** | | | | | | | |
|  | **male** | |  | **female** | |  | **overall** | |
| **Estate** | **n** | **prevalence** |  | **n** | **prevalence** |  | **n** | **prevalence** |
| Alladale | 41 | 12.2a |  | 52 | 5.8a |  | 93 | 8.6b |
| Altnaharra | 32 | 50.0b |  | 34 | 17.6ab |  | 66 | 33.3cd |
| Applecross | 29 | 48.3b |  | 19 | 26.3ab |  | 48 | 39.6d |
| Ardnamurchan | 29 | 24.1ab |  | 25 | 24.0ab |  | 54 | 24.1bd |
| Badanloch | 27 | 14.8ab |  | 35 | 2.9a |  | 62 | 8.1ab |
| Ben Loyal | 33 | 9.1a |  | 21 | 9.5ab |  | 54 | 9.3bc |
| Conaglen | 21 | 28.6ab |  | 18 | 39.9ab |  | 39 | 33.3acd |
| NHT and Aline | 2 | 0.0ab |  | 16 | 56.3b |  | 18 | 50d |
| Strathconon | 28 | 42.9ab |  | 29 | 24.1ab |  | 57 | 33.3cd |
| All | 242 | 27.7 |  | 249 | 18.5 |  | 491 | 23.0 |
|  | **Overall** | | | | | | | |
|  | **male** | |  | **female** | |  | **overall** | |
| **Estate** | **n** | **prevalence** |  | **n** | **prevalence** |  | **n** | **prevalence** |
| Alladale | 74 | 17.6a |  | 100 | 7.0a |  | 174 | 11.5a |
| Altnaharra | 65 | 46.2b |  | 68 | 27.9bc |  | 133 | 36.8b |
| Applecross | 63 | 46.0b |  | 34 | 20.6ac |  | 97 | 37.1b |
| Ardnamurchan | 50 | 30.0b |  | 35 | 20.0ac |  | 85 | 25.9ab |
| Badanloch | 58 | 17.2a |  | 67 | 3.0a |  | 125 | 9.6a |
| Ben Loyal | 66 | 16.7a |  | 60 | 10.0ab |  | 126 | 13.5a |
| Conaglen | 42 | 31.0ab |  | 29 | 41.4c |  | 71 | 35.2b |
| NHT and Aline | 13 | 46.2ab |  | 17 | 58.8c |  | 30 | 53.3b |
| Strathconon | 56 | 41.1ab |  | 62 | 27.4bc |  | 118 | 33.9b |
| All | 487 | 30.8 |  | 472 | 18.4 |  | 959 | 24.7 |
