# Supplementary material for: Prevalence of Liver Fluke (Fasciola hepatica) in Wild Red Deer (Cervus elaphus): Coproantigen ELISA Is a Practicable Alternative to Faecal Egg Counting for Surveillance in Remote Populations
Source: PLoS One. 2016 Sep 6;11(9):e0162420. doi: 10.1371/journal.pone.0162420 (PMC5012657; doi:10.1371/journal.pone.0162420)
Supplement: S1 Text — An explanation of the kappa statistic and its associated equations and interpretational parameters. (DOCX) [file pone.0162420.s008.docx]

**Table S2:** **Example of cross tabulation comparison between two diagnostic methods.**

|  | | Diagnostic method 1 | | |
| --- | --- | --- | --- | --- |
|  |  | **Infected** | **Not infected** | **Total** |
| **Diagnostic** **method 2** | Infected | a | b | g_1_ |
|  | **Not infected** | c | d | g_2_ |
|  | **Total** | f_1_ | f_2_ | N |

$\kappa=\frac{p_{o}-p_{e}}{1-p_{e}}$

**Equation S1:** **(Cohen’s kappa)**

$p_{o}=\frac{a+d}{N}$ proportion of observed agreement between two diagnostics

$p_{e}=\left( \frac{f_{1}}{N} \right)\times\left( \frac{g_{1}}{N} \right)+\left( \frac{f_{2}}{N} \right)\times\left( \frac{g_{2}}{N} \right)$ proportion of expected (chance) agreement

We calculate the proportion of positive agreement as the number of positive diagnoses that are in agreement between both tests as a proportion of the average number of positives recorded by both diagnostic methods combined (**Equation S2**); the proportion of negative agreement is calculated similarly (**Equation S3**).

**(Equation S2)** $p_{\mathrm{pos}}=\frac{a}{\left( \frac{f_{1}+g_{1}}{2} \right)}=\frac{2a}{f_{1}+g_{1}}$

**(Equation S3)** $p_{\mathrm{neg}}=\frac{d}{\left( \frac{f_{2}+g_{2}}{2} \right)}=\frac{2d}{f_{2}+g_{2}}$

Byrt et al., (1993) provides measures of prevalence (PI) and bias (BI) (**Equation S4** and **Equation S5**) and their relation to kappa (**Equation S.6**). Here, the prevalence index (estimated underlying prevalence being the average number of individuals diagnosed as infected between the two methods) and the bias index (underlying bias being a difference in proportions of positive/negative diagnoses between the two methods) are provided (each index ranges 0 to 1; i.e., low to high).

**(Equation S4)** $PI=\frac{2a}{N}-p_{o}=p_{o}-\frac{2d}{N}=\frac{|a-d|}{N}$

**(Equation S5)** $BI=\frac{2b}{N}-1+p_{o}=1-p_{o}-\frac{2c}{N}=\frac{|b-c|}{N}$

**(Equation S6)** $\kappa=\frac{(2p_{o}-{1)-PI}^{2}+\mathrm{BI}^{2}}{1-\mathrm{PI}^{2}+\mathrm{BI}^{2}}$ **kappa in relation to PI and BI**

**Literature cited**

Byrt, T., Bishop, J., Carlin, J.B., 1993. Bias, prevalence and kappa. Journal of Clinical Epidemiology 46, 423–429. doi:10.1016/0895-4356(93)90018-V
